# Genetic and Epigenetic Factors That Predispose to Musculoskeletal Disorders

**DOI:** 10.3390/genes15091194

**Published:** 2024-09-11

**Authors:** Stuart M. Raleigh

**Affiliations:** Centre for Health and Life Sciences, Coventry University, Coventry CV1 5FB, UK; stu.raleigh@gmail.com

Musculoskeletal soft tissue disorders (MSTDs) are a heterogenous group of maladies that can affect the muscles, bones, nerves, joints, ligaments, tendons, cartilage, and adjoining structures and seriously impact on the quality of life in those affected. In some cases, immune system dysregulation is involved (for example, in rheumatoid arthritis), and in other cases, such as sports-related Achilles tendinopathy, there does not seem to be an underlying immune system pathology. The prevalence of these conditions varies widely, depending on the type of disorder and the location affected. For example, the lifetime prevalence of rotator cuff tendinopathy/shoulder pain is around 67% [1], whereas scoliosis affects approximately 8% of the population under 25 years of age, rising to 68% in those over 60 [2]. In this Special Issue, we received five detailed studies that focused predominantly on the molecular genetics of MSTDs and one article that focused on epigenetic factors. We explore these publications in the following paragraphs.

Hassan and colleagues [3] investigated the role of the centriolar protein gene POC5 (POC5) in adolescent idiopathic scoliosis (AIS). They were specifically interested in investigating the role of estradiol (E2) in regulating wild-type POC5 and a mutant version of the gene, POC5^A429V^, in both normal and AIS human osteoblasts, respectively. They used a range of advanced molecular biological techniques in their paper and discovered that POC5 was expressed in a wide range of human tissue, with the highest levels in the pancreas and relatively low levels in the skin and adipose tissue. They also discovered that the A429V mutation was associated with an impaired mineralization rate in AIS cells compared to normal cells and that POC5 was expressed at higher levels in normal osteoblasts compared to AIS osteoblasts that harbored the A429V mutation. They showed that estradiol upregulated POC5 expression in a dose-dependent fashion in normal osteoblasts, but that was not the case in mutant POC5^A429V^ within AIS osteoblasts. Collectively, the study by Hassan and colleagues showed a resistance to the effect of estrogen in AIS, which may have important clinical implications for the management of this condition in the future.

The work of Orchard and co-workers [4] was the only study published in this issue with a focus on epigenetics. Specifically, they investigated histone changes in tissue taken from patients with late-stage rotator cuff tendinopathy (RCT) compared to non-tendinopathic tissue. Using immunoprecipitation sequencing and a range of bioinformatic techniques, they discovered differences in the trimethylation status of H3K4 and H3K27 histones between the RCT tissue and the healthy non-tendinopathic samples. These trimethylation differences may alter the expression of genes and modify the risk of tendinopathy. Interestingly, the work of Orchard and colleagues [4] implicated several genes that have previously been associated with tendinopathy, such as *SMOC2*, *GDF6*, *GDF7*, and TGFβ. Although the authors did not measure gene expression levels in their tissue samples, the work paves the way for a more detailed epigenetic study of RCT in a larger sample of patients affected by the disorder.

Barros and colleagues [5] used a candidate gene association study to investigate the potential role of the rs820218 variant within the SAP30-binding protein (*SAP30BP*) gene and RCT. The authors were keen to establish whether this variant was associated with RTC in an Amazonian population, as previous studies have found an association in other ethnic groups [6]. The study used DNA samples isolated from buccal swabs and qPCR with fluorogenic probes to interrogate the rs820218 locus. The sample size for final genotyping was small (less than 80 in both case and control groups), and the authors did not detect a significant (*p* < 0.05) association between the rs820218 locus and RCT. However, a trend (not statistically significant) between the AA genotype and disease status was observed. Specifically, the AA genotype was only found in 6.7% of the cases but in 20.5% of the controls. Furthermore, the study also revealed an association between both age and hypertension and the risk of RCT. Hence, although the study sample size was modest, this work should encourage additional association studies between the *SAP30BP* gene and RCT in other populations using larger sample sizes.

A larger association study was conducted by Firfirey and colleagues [7] to establish whether variation with the *COMT* and *OPRM1* genes influenced shoulder pain and disability in a cohort of patients having had surgery for breast cancer. This study looked at single locus associations within the two genes, along with haplotypes and allele–allele combinations. Such work has clear implications for the customized treatment of those affected by pain and disability following breast cancer treatment. The study found a number of associations, such as the *COMT* rs4680:A/A genotype being significantly associated with moderate-high pain and pain with disability. They also showed inferred haplotypes within the *COMT* (rs6269 A > G-rs4680 G > A) gene, specifically the G–G and A–A combinations associating with both reduced and increased levels of moderate-high pain, respectively. The authors additionally uncovered a gene–gene interaction effect between combinations of the *COMT* (rs4680 G > A)-*OPRM1* (rs1799971 A > G) and *COMT* (rs4680 G > A)-*OPRM1* (rs540825 T > A) loci that were associated with the reporting of pain and combined (pain and disability) symptoms. These data clearly show a genetic contribution to shoulder pain and disability in South African survivors of breast cancer.

Joint laxity is a complex human phenotype with a known genetic contribution [8]. In a study by Beckley and colleagues [8], a cohort of 106 healthy individuals was used to determine whether polymorphisms within a variety of collagen genes were associated with knee joint laxity measurements and ligament length changes within the non-dominant leg. The authors revealed significant associations between combinations of the *COL5A1* rs12722 CC, *COL11A1* rs3753841 CC, *COL11A1* rs1676486 TT, and *COL11A2* rs1799907 AA genotypes and knee hyperextension in their cohort. They also showed that male sex, age, and body mass impacted knee laxity measurements. This study highlights the fact that genotype combinations (within the measured loci) affect anatomical functions and that this may be important in predicting the risk of sports-related injury or, indeed, the risk of musculoskeletal issues in an aging population.

The use of fluoroquinolone antibiotics, such as ciprofloxacin (CPX), is known to be associated with an increased risk of tendinopathy [9]. However, a detailed understanding of the effects of CPX on tendon matrix enzymes and proteoglycan catabolism is incomplete and was the subject of the study submitted to this Special Issue by James and co-workers [10]. In their study, they used equine tendon explant cultures to study the effect of various concentrations of CPX on proteoglycans, aggrecan, versican, and a range of matrix-degrading enzymes. The authors found that CPX prevented the loss of proteoglycans in a dose independent manner. They also showed that the CPX-induced repression of proteoglycan breakdown was paralleled by a downregulation of specific MMP and ADAMPTS mRNAs. This study used an extensive array of cell culture and molecular biological techniques to investigate the effect of CPX on the tendon explants, and it significantly adds to our understanding of CPX-related tendinopathies.

In summary, the studies submitted to this Special Issue are of high quality and advance our understanding of the pathologies studied. The papers span a spectrum of musculoskeletal problems, including AIS, tendinopathy, and pain/disability associated with breast cancer treatment. The research focus was predominantly on genetic factors associated with these conditions and only one of the papers [4] focused on epigenetic factors. As the editor of this Special Issue, I hope the work presented here will stimulate additional studies in these areas and lead to new discoveries and better outcomes for those affected.
